# Dystonia-like behaviors and impaired sensory–motor integration following neurotoxic lesion of the pedunculopontine tegmental nucleus in mice

**DOI:** 10.3389/fneur.2023.1102837

**Published:** 2023-03-30

**Authors:** Jun-Hui Su, Yao-Wen Hu, Yun-Ping Song, Yi Yang, Ruo-Yu Li, Kai-Ge Zhou, Ling Hu, Xin-Hua Wan, Fei Teng, Ling-Jing Jin

**Affiliations:** ^1^Department of Neurology, Shanghai Tongji Hospital, School of Medicine, Tongji University, Shanghai, China; ^2^Department of Neurology and Neurological Rehabilitation, Shanghai Yangzhi Rehabilitation Hospital (Shanghai Sunshine Rehabilitation Center), School of Medicine, Tongji University, Shanghai, China; ^3^Department of Laboratory Animal Science, Fudan University, Shanghai, China; ^4^Department of Neurology, Peking Union Medical College Hospital, Chinese Academy of Medical Sciences, Beijing, China

**Keywords:** pedunculopontine nucleus, dystonia, sensory-motor integration, motor performance, reticular formation, striatal pathways

## Abstract

**Introduction:**

The pedunculopontine nucleus (PPTg) is a vital interface between the basal ganglia and cerebellum, participating in modulation of the locomotion and muscle tone. Pathological changes of the PPTg have been reported in patients and animal models of dystonia, while its effect and mechanism on the phenotyping of dystonia is still unknown.

**Methods:**

In this study, a series of behavioral tests focusing on the specific deficits of dystonia were conducted for mice with bilateral and unilateral PPTg excitotoxic lesion, including the dystonia-like movements evaluation, different types of sensory-motor integrations, explorative behaviors and gait. In addition, neural dysfunctions including apoptosis, neuroinflammation, neurodegeneration and neural activation of PPTg-related motor areas in the basal ganglia, reticular formations and cerebellum were also explored.

**Results:**

Both bilateral and unilateral lesion of the PPTg elicited dystonia-like behaviors featured by the hyperactivity of the hindlimb flexors. Moreover, proprioceptive and auditory sensory-motor integrations were impaired in bilaterally lesioned mice, while no overt alterations were found for the tactile sensory-motor integration, explorative behaviors and gait. Similar but milder behavioral deficits were found in the unilaterally lesioned mice, with an effective compensation was observed for the auditory sensory-motor integration. Histologically, no neural loss, apoptosis, neuroinflammation and neurodegeneration were found in the substantia nigra pars compacta and caudate putamen (CPu) following PPTg lesion, while reduced neural activity was found in the dorsolateral part of the CPu and striatal indirect pathway-related structures including subthalamic nucleus, globus pallidus internus and substantia nigra pars reticular. Moreover, the neural activity was decreased for the reticular formations such as pontine reticular nucleus, parvicellular reticular nucleus and gigantocellular reticular nucleus, while deep cerebellar nuclei were spared.

**Conclusion:**

In conclusion, lesion of the PPTg could elicit dystonia-like behaviors through its effect on the balance of the striatal pathways and the reticular formations.

## Introduction

Dystonia is a movement disorder featured by consistent and intermittent muscle contraction, leading to repetitive involuntary movements and twisting postures (1). Dystonia patients often present with dystonic movements or/and postures (mainly flexion), along with impairments in sensory–motor processing, and their quality of life is severely affected (2). Classically, dystonia is a network disorder of the basal ganglia (BG) characterized by an imbalance between the direct and indirect pathway and the disinhibition of the motor cortex appears following the reduced discharge of BG output nuclei such as the globus pallidus internus (GPi) and substantia nigra pars reticulata (SNpr) (3). With evidence showing that the cerebellum has connected with the BG and is closely correlated with dystonia, the impairment of the BG-cerebello-thalamo-cortical circuit is proposed for the pathophysiological fundamental of dystonia, and the dysfunction of the dopaminergic and cholinergic systems in this circuit was noted (4). Within this circuit, the pedunculopontine tegmental nucleus (PPTg) is a vital interface between the BG and the cerebellum, which might participate in locomotion and muscle tone control (5).

The PPTg is located in the dorsal pontomesencephalic tegmentum with direct connections with the BG and cerebellum, and it is a major component of the mesencephalic locomotor region with functions in motor initiation, muscle tone, and speed (6–8). Neuropathological changes of the PPTg are found in many types of movement disorders such as Parkinson's disease and progressive supranuclear palsy (9). In autopsy studies on patients with isolated dystonia, neurofibrillary tangles, cholinergic neural loss, and perinuclear inclusions of the PPTg were reported (10, 62), and these pathological changes were also found in patients of dystonia combined with parkinsonism (11). In a transgenic DYT1 mouse model of dystonia, dystonia-like behaviors including self-clasping of limbs, abnormal head posture, and circling were observed, and perinuclear aggregates and inclusions were found in the PPTg (12). In addition, significant cholinergic deficits were found for the PPTg in a Dst^dt−J^ mouse model of dystonia, and mice presented obvious dystonia movements along with severe spastic ataxia, imbalance, and body tremors (13). In addition to dystonic-like behaviors, genetic dystonia models showed deficits in skilled motor tasks and fine motor control, such as decreased latency to fall in the accelerated rotarod and increased slip numbers in the beam-walking test (14). Tactile, proprioceptive, and nociceptive sensory–motor integrations were also impaired in patients and the mouse model of dystonia (15, 16).

Until now, preclinical studies have reported that a bilateral non-specific lesion of the PPTg could induce the increasement of muscle tone, impairment of limb use, and righting reflex in rats (17). It is also reported that a bilateral excitotoxic lesion of the PPTg could induce persistent impairments on accelerated rotarod and fine motor control (18). Moreover, a bilateral lesion of the PPTg could reduce the level of prepulse inhibition (PPI) of the acoustic startle response, which indicates that the PPTg is involved in the sensory–motor gating (19). According to the results of neuromodulation studies, the electrical stimulation of the PPTg could suppress the muscle tone of hindlimbs in decerebrated cats, and optogenetic stimulation of different neural populations of the PPTg could evoke motor responses of flexor and extensor muscles of hindlimbs in mice (20, 21). Although increasing evidence showed the potential role of the PPTg in locomotion and muscle tone control, there is a lack of evidence demonstrating whether manipulating the PPTg might induce dystonia-like behaviors and dystonia-related sensory–motor dysfunction. In addition, dystonia is featured by the imbalance of the striatal direct and indirect pathways, and the reduced striatal indirect pathway might result in the loss of inhibition (22). With abundant anatomical connections between the PPTg and the BG-cerebello-thalamo-cortical circuit, whether impariments of the PPTg could mimic the dystonia-related circuit dysfunction is also under exploration.

Based on the previous findings of the abnormal behaviors and impaired sensory–motor functions in mouse models of dystonia, a series of sensitive behavioral tests including specific dystonia evaluations, different types of sensory–motor integrations, explorative behaviors, and gait were conducted for mice under a bilateral and unilateral PPTg excitotoxic lesion. In addition, neural dysfunctions such as apoptosis, neuroinflammation, neurodegeneration, and the neural activation of dystonia-related motor areas in the BG, reticular formations, and cerebellum were also explored in this study.

## Materials and methods

### Experimental subject

Adult (25–30 g; 12–16 weeks old) male C57BL/6N mice (Beijing Vital River Laboratory Animal Technology Co., Ltd.) were used in the experiments. All mice were bred in a humidity- and temperature-controlled animal facility. Lights were on for 12 h per day (from 7:00 to 19:00). A total of four mice were housed in each cage and had free access to water. Behavioral tests were carried out during light-on periods. All experiments were approved by the Institutional Animal Care and Use Committee of Tongji University and conducted in accordance with the ethical health guide for the care and use of laboratory animals (TJBC00320101).

### Stereotaxic surgery

Each mouse was intraperitoneally anesthetized with 0.8% pentobarbital sodium (Sigma-Aldrich, St. Louis, MO, USA) under a dosage of 93 mg/kg. Once anesthetized, the head was secured in a stereotaxic frame (RWD Life Science, Shenzhen, China), and the skull was exposed with a midline incision. Thereafter, a perforation was made with a cranial drill to allow the toxin infusion at the appropriate coordinates below. After the infusion, the incision was sutured, and the animal was placed in a heated pad until full recovery.

### Excitotoxic lesions

Ibotenic acid (IBO; MedChemExpress, Monmouth Junction, NJ, USA) was used as excitotoxin with a dose of 5 mg/mL [31.62 mM; dissolved in sterile phosphate buffer solution (PBS); pH 7.4]. IBO was injected into the bilateral PPTg [anterior-posterior (AP) −4.60 mm; medial-lateral (ML): ±1.20 mm; dorsal-ventral (DV): −3.70 mm] or the unilateral PPTg [right side; AP: −4.60 mm; ML: +1.20 mm; DV: −3.70 mm] through a glass micropipette, with 150 nL per side at a speed of 10 nL/min. After each injection, the micropipette was left for an additional 15 min before being slowly withdrawn. Sham mice underwent an identical volume of PBS injected instead of IBO. The stereotaxic coordinates for microinjection referred to Paxinos and Franklin's mouse brain in stereotaxic coordinates (23). The dose and volume of IBO for mice referred to previous rat studies of PPTg IBO lesions (19, 24).

### Behavioral tests

**Figure 1:**
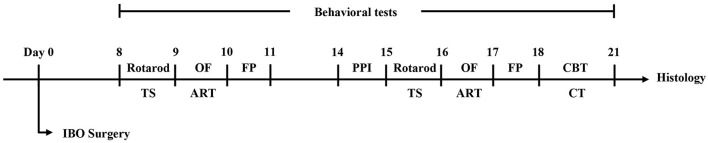
The flowchart of experimental design. TS, tail suspension test; OF, open field test; ART, adhesive removal test; FP, footprint test; PPI, prepulse inhibition test; CBT, challenge beam test; CT, cylinder test.

All behavior tests were carried out in a soundproof room with controlled temperature, light, and humidity. The tests were all conducted during the light phase of the light/dark cycle. The mice were acclimated for 2 h in the behavioral room before training and testing. According to the finding of previous studies that an IBO lesion was pathologically significant since day 7 (25, 26), day 7 and day 14 were chosen for evaluated the abnormal behavior of the mice. The experimental design is shown in Figure 1.

#### Scoring system for dystonia-like behaviors

Dystonia behaviors were evaluated through the tail suspension test and the cylinder test. According to earlier reports that many dystonia behaviors might be prominent following stress and fatigue exposure (27), the tail suspension test was performed 30 min after the rotarod test. Each mouse was picked up by the tail near the base and recorded in front of a high-speed camera for 2 min. Videos were reanalyzed by two blinded experimenters (J-HS and Y-WH) based on an 8-point dystonia scoring system (28). For forelimb assessment, sustained or repeated tonic flexions and retractions as well as hyperextension and crossing were considered dystonia-like, and a total of four points were scored: (0) no abnormal movements, (1) decreased movement of forelimbs with hyperextension of paws occurred by ≥50% of the recorded time; (2) mild dystonia-like movements of the forelimb(s) of <50% of the recorded time, (3) mild dystonia-like movements of the forelimb(s) of ≥50% of the recorded time or severe movements of < 50% of the recorded time, and (4) severe dystonia-like movements of the forelimb(s) of ≥ 50% of the recorded time. For hindlimb assessment, retraction, clenching, and clasping of hindlimbs as well as sustained hyperextension were considered dystonia-like movements, and a total of three points were scored: (0) no abnormal movements, (1) decreased movement of hindlimbs with hyperextension of paws seen by ≥ 50% of the recorded time, (2) dystonia-like movements of one hindlimb, and (3) dystonia-like movements of both hindlimbs. For trunk assessment, a truncal distortion of >80% of the recorded time was scored for an additional 1 point.

In addition, the cylinder test was used for the evaluation of the spontaneous dystonia-like posture of the mouse. A mouse was placed in a glass cylinder (9.5 cm O.D, 17.5 cm height) and recorded for 5 min as an earlier report (29). The frequency and distribution of dystonia-like movements were evaluated based on a 4-point scale similar to clinical rating scales for dystonia (28): (0) normal behavior, (1) abnormal motor behavior without dystonia-like movements, (2) mild motor impairment with mild focal dystonia-like movements, (3) moderate motor impairment with severe focal dystonia-like movements, and (4) severe impairment with sustained and generalized dystonia-like movements.

#### Challenge beam test

A modified beam test was conducted for measuring skilled motor function and proprioceptive sensory–motor integrity during spontaneous walking. The plexiglass beam consisted of four segments (25 cm each) with different widths (from 3.5 to 1 cm, with 1 cm decrements each). The mice were trained to traverse the beam from the widest segment to the narrowest for 2 days, with five trials per day. During the first two trials, the mice were assisted to traverse the beam with the guidance of a home cage placed in proximity to them. On day 3 of testing, a mesh grid (1 cm squares) of corresponding width was placed over each beam with a height of 1 cm, and the mice were placed on the widest mesh grid to traverse over the whole beam during videotaped.

The videos were reassessed in slow motion by a blinded experimenter (JHS). The errors and steps were counted for each trial, and an average ratio of errors and steps was calculated. An error was defined as the limb slipping beyond 0.5 cm below the grid surface. A detailed process of evaluation is given in an earlier report (30).

#### Rotarod test

The passively proprioceptive sensory–motor integration and motor learning were also assessed through the accelerating rotarod test. For training, the mice were placed on the running rod (Ugo Basile SRL, Italy) at the constant speed of 5 rpm for 1 min, and a total of three sets of training were performed with an interval of 15 min. Thereafter, testing was conducted in accelerating mode with speed accelerating from 4 to 40 rpm in 300 s. Each mouse received 3–4 tests with an interval of 15 min. The latency to fall off the apparatus was recorded.

#### Adhesive removal test

The adhesive removal test was performed to evaluate tactile sensory–motor integration ability, and a delay in touching and removing the sticker on the snout was defined as impaired. In this test, the mice were gently restrained for 3–5 s until no struggling presented. Thereafter, a round sticker (3 mm in diameter) was pasted on the snout, and the mice were immediately returned to the cage with only the bedding material but without other littermates. Meanwhile, another experimenter (YY) recorded the latency to touch and remove the sticker. Each mouse underwent three consecutive trials with an interval of 10 min, and the average time to touch and removal was calculated and compared.

#### Prepulse inhibition

The auditory sensory–motor integration was evaluated through PPI. PPI was conducted through sound attenuating test chambers (65 × 35 × 25 cm, *L* × *W* × *H*), and a startle reflex system (SR lab, San Diego, CA, USA) was used for testing. Once the mouse was restrained in the chamber, a 5-min acclimation of 50-dB acoustic background white noise was initiated. After the acclimation, 12 trials of 120 dB startle stimuli (40 ms; square wave) with an interval of 15 s were performed for the evaluation of the acoustic startle reflex. Thereafter, either startle stimuli alone or after three different prepulse intensities (65, 73, and 85 dB, 20 ms) with a delay of 100 ms were randomly performed in the sequential 48 trials, with a mean intertrial interval of 15 s. The percentage of PPI of different prepulse stimulus intensities was calculated through the equation: PPI = 100 – [(prepulse/startle alone) × 100].

#### Open field

The open field test was conducted for evaluating the spontaneous explorative activity. Each mouse was initially placed in a random corner of the open field apparatus (Med Associates, Inc., Phoenix, Arizona, USA). The apparatus contained a transparent plexiglass box (27.31 × 27.31 × 21 cm, *L* × *W* × *H*) which was in a soundproof box with lighting control (200 lx). Mice were allowed to freely explore the box for 30 min. Ambulatory counts/distance/average speed, jump counts, stereotypic counts and vertical counts were recorded by Activity Monitor software (Med Associates, Inc., Phoenix, Arizona, USA).

#### Footprint test

The footprint test was conducted for gait assessment. The feet of the mouse were painted with non-toxic washable paint, and the mouse was allowed to walk through a tunnel (33.02 × 6.35 × 7.62 cm, *L* × *W* × *H*) on a sheet of paper. Before testing, the mice were allowed to walk through the tunnel until naturally walking forward. A total of three clear sequential prints of forelimbs and hindlimbs were chosen for assessment. The stride length, width, and toe spread were recorded and analyzed according to the previous study (31).

### Animal sacrifice and tissue processing

The mice were intraperitoneally anesthetized with 0.8% pentobarbital sodium and perfused with 0.1 M PBS followed by 4% paraformaldehyde. Thereafter, the brains were dissected and post-fixed in 4% PFA at 4°C overnight. After fixation, the brains were cryoprotected in 20% sucrose for 1 day, followed by 30% sucrose for 2 days at 4°C before being refrigerated at −80°C. The brains were sectioned on a freezing cryostat (CM1950, Leica) at 30 μm into six series, and each series was mounted on glass slides and used for further experiments.

### Immunofluorescence

For immunofluorescence, brain slices were first treated with sodium citrate (0.01 M, pH 6.0) at 95°C for 10 min. After being rinsed with PBS for three times (each for 15 min), brain sections underwent blocking with serum containing 0.5% Triton X-100 for 2 h at room temperature, followed by primary antibody incubation at 4°C overnight. The following primary antibodies were used: goat anti-ChAT (1:500; AB144P; Sigma-Aldrich), rabbit anti-NeuN (1:500; ab177487; Abcam), rabbit anti-Glial fibrillary acidic protein (GFAP; 1:1000; Z0334; Dako), rabbit anti-Cleaved caspase-3 (1:500; 9661s; Cell signaling technology), rabbit anti-Iba1 (1:200; A19776; Abclonal), rabbit anti-alpha-synuclein (phosphor S129) (1:500; EP1536Y; Abcam), mouse anti-Tyrosine hydroxylase (TH; 1:1000; T2928; Sigma-Aldrich), and mouse anti-c-Fos (1:100; sc-166940; Santa Cruz). After primary antibodies incubation, all sections were rinsed with PBS three times (each for 15 min) and stained with species-specific Alexa Fluor 488- or biotin- conjugated secondary antibodies at room temperature for 3 h. Thereafter, Cy3-conjugated streptavidin and Hoechst 33,258 were used for biotin-conjugated secondary antibodies for 1 h at room temperature, and all sections were mounted with 75% glycerol.

### Stereological neuronal counts

Images of the BG, PPTg, reticular formations, and cerebellar structures were captured on a fluorescence microscope (Eclipse 80i, Nikon). A 10x objective lens was used for imaging collection, and a blinded investigator performed cell counts using the ImageJ software. Lesioned areas of the PPTg were drawn on schematic sections of an electronic stereotaxic atlas according to Paxinos and Franklin's Mouse Brain in Stereotaxic Coordinates (23) based on the NeuN staining. Every lesion was drawn and overlaid onto the schematic sections throughout the rostro-caudal extent of the PPTg from AP −4.38 mm to AP −5.10 mm. Composite images of different layers of the PPTg were formed to show the lesioned areas.

Because cholinergic neurons are specifically expressed throughout the rostro-caudal extent of the PPTg and outlined its boundary, the counting of cholinergic neurons was adapted to show the degree of the lesion (26, 32). The location of the PPTg at the midbrain level was confirmed laterally to the superior cerebellar peduncle. The border of the PPTg was confirmed according to the fluorescence staining of ChAT. The absolute numbers of cholinergic neurons on both sides of the anterior (at APs −4.20 mm and −4.38 mm), central (at APs −4.56 mm and −4.74 mm) and posterior PPTg (at APs −4.92 mm and −5.10 mm) were counted. Moreover, the absolute number of cholinergic neurons in the laterodorsal tegmental nucleus (LDT) which is located caudally of the PPTg were also counted for layers of APs of −5.10 mm, −5.28 mm, 5.46 mm, and −5.60 mm. In addition, the fluorescence signal intensity was quantified for GFAP and Iba1 responses in the PPTg at an AP of −4.74 mm through the ImageJ software.

Dopaminergic neurons in the substantia nigra pars compact (SNpc) were identified by their specific marker TH. Cell counting was performed on dopaminergic neurons in the bilateral SNpc at an AP of −2.79 mm, −2.97 mm, −3.15 mm, and −3.33 mm, respectively. In addition, cell counting was also performed for cholinergic neurons in the caudate putamen (CPu), which is given in an earlier report (33). Since the dorsal side of the CPu was mainly involved in the selection of movement, the AP +0.13 mm layer of the striatum was selected, and the CPu was divided into dorsolateral, dorsomedial, ventrolateral, and ventromedial regions for counting cholinergic neurons, respectively. In addition, the c-Fos+ cells were counted in bilateral BG, including the dorsolateral part of the CPu, globus pallidus externus (GPe; at APs of −0.23 mm, −0.41 mm, and −0.59 mm), GPi (also called entopeduncular nucleus in mice; at APs of −1.23 mm, and −1.41 mm), subthalamic nucleus (STN; at APs of −1.94 mm, −2.02 mm, and −2.20 mm), SNpc and SNpr (at APs of −2.61 mm, −2.79 mm, and −2.97 mm). Reticular formations and cerebellar structures which had connections with the PPTg were also counted for c-Fos+ cells, including the bilateral pontine reticular nucleus oral part (PnO; at APs of −4.56 mm, 4.74 mm, and −4.92 mm), caudal part (PnC; at APs of −5.10 mm, −5.28 mm, −5.46 mm, and −5.64 mm), parvicellular reticular nucleus (PCRt; at APs of −6.36 mm, −6.54 mm, and −6.72 mm), gigantocellular reticular nucleus (Gi; at APs of −6.36 mm, −6.54 mm, and −6.72 mm), interposed cerebellar nucleus anterior part (IntA; at APs of −6.00 mm and −6.18 mm), and lateral cerebellar nucleus (Lat; at APs of −6.00 mm and −6.18 mm).

### Statistical analysis

Statistical analysis was conducted using SPSS version 21.0 (IBM Inc.), and plots were drawn using GraphPad Prism 9 (GraphPad software). Normally distributed data were shown as means ± SEM, and Student's *t-*test was used for comparison between the experimental and control group. The Mann–Whitney *U*-test was used if the data did not follow a normal distribution. A *P-*value of < 0.05 was defined as statistically significant.

## Results

### Confirmation of IBO lesion of the PPTg

**Figure 2:**
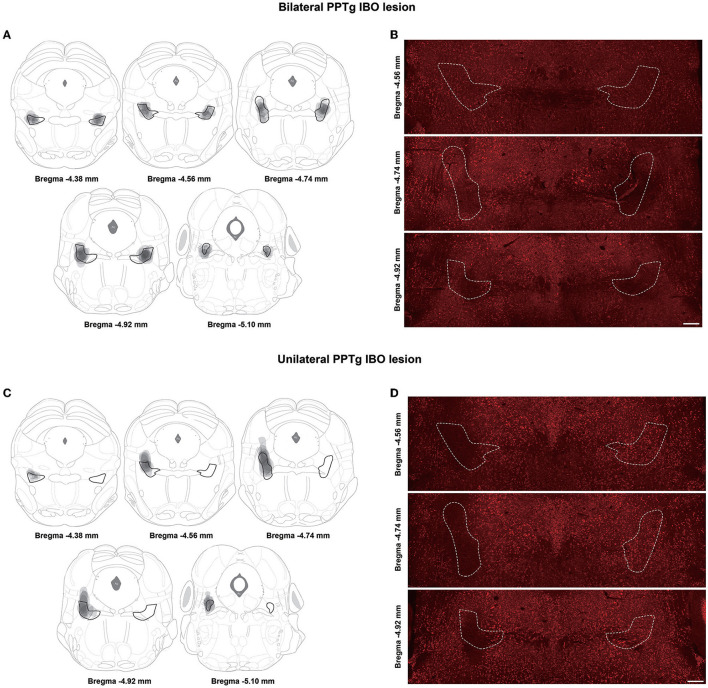
Lesioned areas in different layers of the PPTg for bilateral and unilateral PPTg IBO lesioned mice. **(A)** The overlay of lesioned areas in the bilateral PPTg IBO lesioned group is drawn according to the neuronal nuclei (NeuN) staining. The PPTg is outlined in black and every lesioned area is drawn in gray. **(B)** Representative photographs of NeuN staining in different layers of the PPTg for bilateral IBO lesioned mice. The PPTg is outlined in the dashed line. **(C)** The overlay of lesioned areas in the unilateral PPTg IBO lesioned group is drawn according to the NeuN staining. **(D)** Representative photographs of NeuN staining in different layers of the PPTg for unilateral IBO lesioned mice. Scale bar = 1000 μm.

Based on the results of NeuN staining and cholinergic neuron counting covering all the extent of PPTg, eight mice were confirmed as successfully having bilateral PPTg lesions, while seven mice had unilateral PPTg (right side) lesions. Relatively, seven mice had sham lesions on the bilateral PPTg and seven mice had on the right PPTg. Composite images of different layers of the PPTg with overlays of lesioned areas in all bilaterally and unilaterally lesioned mice are shown in Figures 2A, C. The mice with a bilateral PPTg lesion had their lesion restricted and covered throughout all extents of the PPTg, while the mice with a unilateral PPTg lesion had their lesion distributed slightly dorsal in the central PPTg (Figures 2B, D).

**Figure 3:**
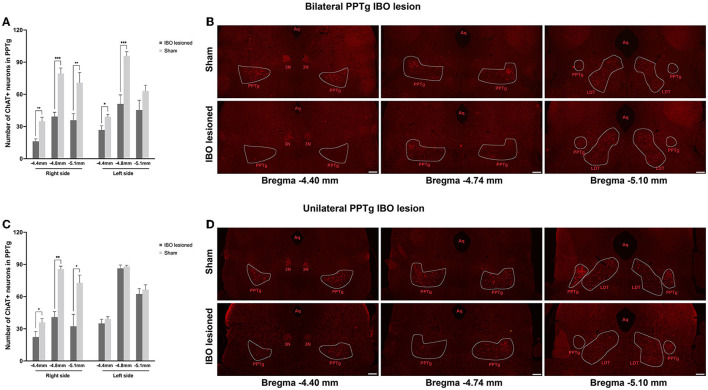
Quantification of cholinergic neurons and lesion analysis in different layers of the PPTg for mice that underwent bilateral and unilateral PPTg IBO lesion. **(A)** The comparison of the absolute number of cholinergic neurons in the anterior (~AP −4.4 mm), central (~AP −4.8 mm), and posterior (~-5.1 mm) PPTg between the bilateral IBO lesioned and sham lesioned mice. **(B)** Representative photographs of ChAT staining of the anterior, central, and posterior PPTg for the bilateral IBO and sham lesioned mice. **(C)** The comparison of the absolute number of cholinergic neurons in the anterior (~AP −4.4 mm), central (~AP −4.8 mm), and posterior (~-5.1 mm) PPTg between unilateral IBO lesioned and sham lesioned mice. **(D)** Representative photographs of ChAT staining of anterior, central, and posterior PPTg for unilateral IBO and sham lesioned mice. The PPTg is outlined with the dotted line. *P < 0.05, **P < 0.01, ***P < 0.001. Scale bar = 1000 μm.

For bilateral PPTg IBO-lesioned mice, the number of cholinergic neurons was robustly reduced for the anterior (lesioned: 16.13 ± 2.40, sham: 34.86 ± 3.74; *P* = 0.001), central (lesioned: 39.25 ± 4.09, sham: 79.43 ± 5.10; *P* < 0.001), and posterior PPTg (lesioned: 35.88 ± 6.06, sham: 70.86 ± 9.49; *P* = 0.007) on the right side, nearly to 50%. In addition, cholinergic neurons were significantly lost in the left anterior (lesioned: 26.75 ± 3.93, sham: 39.00 ± 2.41; *P* = 0.024) and central PPTg (lesioned: 51.13 ± 8.34, sham: 95.86 ± 3.85; *P* < 0.001), while an insignificant reduction of cholinergic neurons in the posterior PPTg was found compared with the sham group (lesioned: 45.38 ± 9.17, sham: 63.14 ± 5.30; *P* = 0.131). The cholinergic neuron loss and quantification in different layers of the PPTg are shown in Figures 3A, B. For the unilateral PPTg lesioned mice, significant loss of cholinergic neurons was found in the anterior (lesioned: 22.29 ± 4.97, sham: 35.86 ± 3.68; *P* = 0.049), central (lesioned: 40.86 ± 5.10, sham: 85.57 ± 2.80; *P* = 0.001, Mann-Whitney *U* test), and posterior PPTg (lesioned: 32.29 ± 11.26, sham: 72.86 ± 7.13; *P* = 0.010) on the right side, while the left PPTg remained intact. The quantification of cholinergic neurons and lesion analysis in different layers of the PPTg are shown in Figures 3C, D. Moreover, both bilaterally and unilaterally lesioned mice showed enhanced astroglial and microglial responses nearby the infusion area compared with the sham mice (Supplementary Figure S1).

Since the LDT also contained cholinergic neurons and was located caudally to the PPTg, in order to confirm that the lesion was restricted in the PPTg, the quantification of cholinergic neurons was performed for LDT. In the results, there was no significant change of cholinergic neurons in the entire extent of the LDT for bilaterally and unilaterally lesioned mice (Supplementary Figure S2), indicating that the LDT was not affected by the lesion.

### Dystonia-like behaviors following the IBO lesion of the PPTg

**Figure 4:**
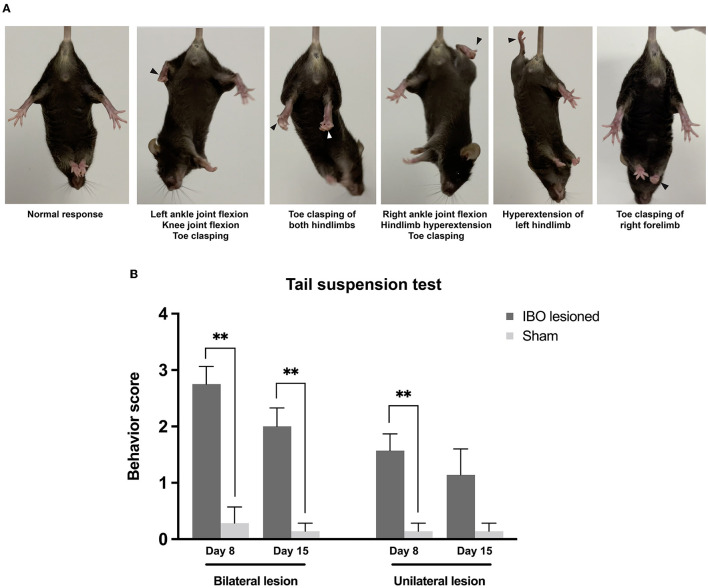
Dystonia-like behaviors following bilateral and unilateral PPTg IBO lesion. **(A)** Photographs of representative dystonia-like behaviors in mice following a bilateral and unilateral PPTg IBO lesion. Arrows indicated abnormal behaviors. **(B)** Dystonia score of the tail suspension test in mice that underwent a PPTg IBO lesion. Both the bilaterally and unilaterally PPTg IBO lesioned mice had higher dystonia scores after the lesion on day 8, and a significant difference persisted in the bilaterally lesioned group on day 15. *P < 0.05, **P < 0.01, ***P < 0.001.

After a week of injecting the IBO into the PPTg, both bilaterally and unilaterally lesioned mice showed overt dystonia-like behavior while suspended, and these behaviors persisted until the second evaluation at 2 weeks. Dystonia-like behaviors mainly appeared in the hindlimbs, which primarily presented as flexion of ankle and knee joints, accompanied by sustained toe clasping (Figure 4A and Supplementary Video 1). For bilaterally lesioned mice, 7/8 of them had toe clasping of the hindlimb, with 4/7 of them bilaterally affected. In addition, 4/8 of mice presented flexion of knee and ankle joints, and all mice had hyperextension of hindlimbs. For unilaterally lesioned mice, 5/7 of them had unilateral toe clasping, but no mice showed flexion of knee and ankle joints. Moreover, 3/7 of them showed hyperextension of the hindlimbs. The dystonia score for mice that underwent bilateral PPTg lesions was significantly higher than that of the bilateral sham group at 1 week (lesioned: 2.75 ± 0.25, sham: 0.29 ± 0.29; *P* = 0.001, Mann–Whitney *U*-test) and 2 weeks after the lesion (lesioned: 2.00 ± 0.33, sham: 0.14 ± 0.14; *P* = 0.001, Mann–Whitney *U*-test). For the mice that underwent the unilateral PPTg lesion, the dystonia score at 1 week was significantly higher than the sham group (lesioned: 1.57 ± 0.30, sham: 0.14 ± 0.14; *P* = 0.002, Mann-Whitney *U*-test), but it became insignificant at 2 weeks after the injection (lesioned: 1.14 ± 0.46, sham: 0.14 ± 0.14; *P* = 0.128, Mann–Whitney *U*-test). With the recovery duration prolonged, there was an alleviation of the dystonia-like behaviors in both the lesioned groups. However, bilaterally lesioned mice still showed severe dystonia regardless of recovery (Figure 4B), and detailed scoring for each mouse is shown in Supplementary Table S1. While dystonia-like behaviors appeared for PPTg lesioned mice during the suspension test, there were no overt dystonia manifestations for these mice during spontaneous locomotion in the cylinder.

### Impairments of skilled motor function and sensory–motor integration after the PPTg lesion

**Figure 5:**
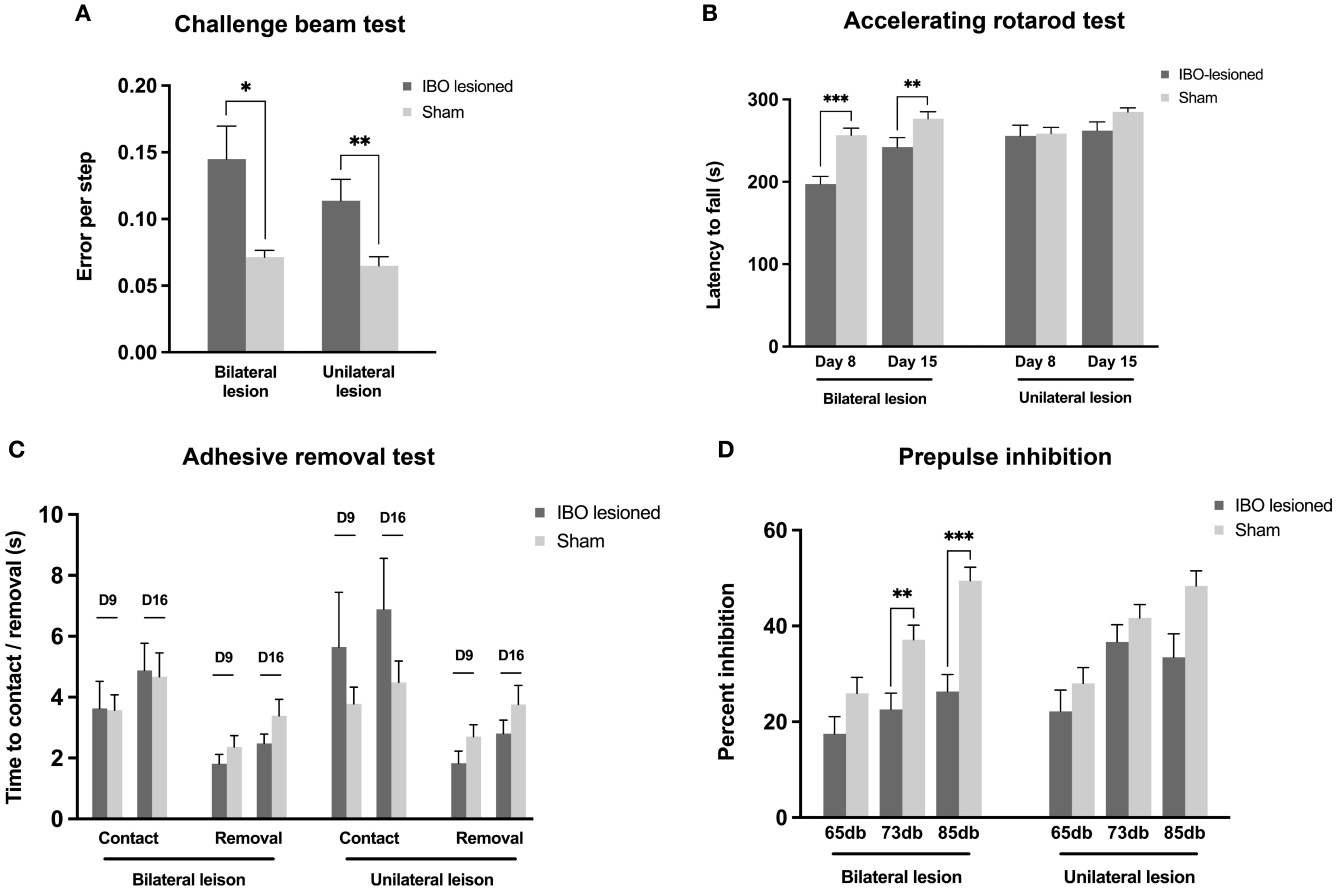
Impaired skilled motor functions and sensory–motor integration following PPTg IBO lesioned. **(A)** Both the bilaterally and unilaterally PPTg IBO lesioned mice showed elevated error per step in the challenge beam test compared with sham mice. **(B)** Significantly reduced latency to fall was seen in bilateral PPTg IBO lesioned mice at day 8 and day 15, while no difference was found between unilateral lesioned and sham mice in accelerating rotarod test. **(C)** No difference was found for time to contact and removal of the adhesive sticker on the snot between PPTg lesioned and sham mice at day 9 and day 16. **(D)** Decreased inhibition of prepulse stimulation was found in the bilateral PPT IBO lesioned mice at 73 db and 85 db, while no difference was seen between unilateral PPTg lesioned and sham group. *P < 0.05, **P < 0.01, ***P < 0.001.

Both bilateral (lesioned: 0.14 ± 0.02, sham: 0.07 ± 0.01; *P* = 0.022) and unilateral PPTg lesioned mice (lesioned: 0.12 ± 0.02, sham: 0.06 ± 0.01; *P* = 0.010) showed significantly increased errors per step in the challenge beam test (Figure 5A). In accelerating the rotarod test, bilaterally lesioned mice showed reduced latency to fall at day 8 (lesioned: 197.26 ± 9.33 s, sham: 256.68 ± 8.71 s; *P* < 0.001) and day 15 after the lesion (lesioned: 242.33 ± 11.63 s, sham: 276.60 ± 8.49 s; *P* = 0.008, Mann–Whitney *U*-test), while unilaterally lesioned mice showed no abnormality (Figure 5B). In addition, the latency to fall increased in both lesioned groups at the second evaluation point compared with the first, indicating that the motor learning ability was retained for these mice (Figure 5B). In the adhesive removal test, PPTg-lesioned mice spent comparable time contacting the sticker on the snout, and the contact was immediately followed by the removal, which reflects that there was no overt impairment on the facial tactile sensory–motor integration and fine motor control (Figure 5C). Regarding the auditory sensory–motor integration, both bilaterally (lesioned: 33.80 ± 2.12, sham: 42.21 ± 4.10; *P* = 0.081) and unilaterally lesioned group (lesioned: 34.63 ± 3.54, sham: 41.18 ± 3.69; *P* = 0.224) had comparable startle response than the sham group. However, bilaterally lesioned mice showed significantly decreased percent inhibition at 73 db (lesioned: 22.60 ± 3.39, sham: 37.11 ± 3.06; *P* = 0.005, Mann–Whitney *U*-test) and 85 db (lesioned: 26.35 ± 3.54, sham: 49.48 ± 2.80; *P* < 0.001, Mann–Whitney *U-*test), while unilaterally lesioned mice had comparable percent inhibition than the sham mice (Figure 5).

### PPTg IBO lesion did not cause overt deficits in spontaneous exploration and gait

In the open field test, bilaterally lesioned mice showed decreased average ambulatory episode speed at day 16 compared to the sham group (lesioned: 27.78 ± 1.41 cm/s, sham: 34.45 ± 2.12 cm/s; *P* = 0.019; Supplementary Figure S3B), and the vertical counts were also reduced at day 9 (lesioned: 143.25 ± 15.58, sham: 235.57 ± 29.25; *P* = 0.034; Supplementary Figure S3E). However, these differences were not seen in mice under the unilateral PPTg IBO lesion. There was no difference in ambulatory distance, ambulatory counts, stereotypic counts, and jump counts for both bilaterally and unilaterally lesioned mice when compared with the sham mice (Supplementary Figure S3). With regard to the gait, bilaterally lesioned mice showed shorter stride length and higher stride width for the hindlimbs, but no significant difference was found (Supplementary Figures S4A–C). In addition, there was no difference between unilaterally lesioned mice and the sham mice in terms of the stride length, width, and toe spread (Supplementary Figures S4D–F).

### PPTg IBO lesion did not induce apoptosis and neuron loss in the SNpc and CPu

**Figure 6:**
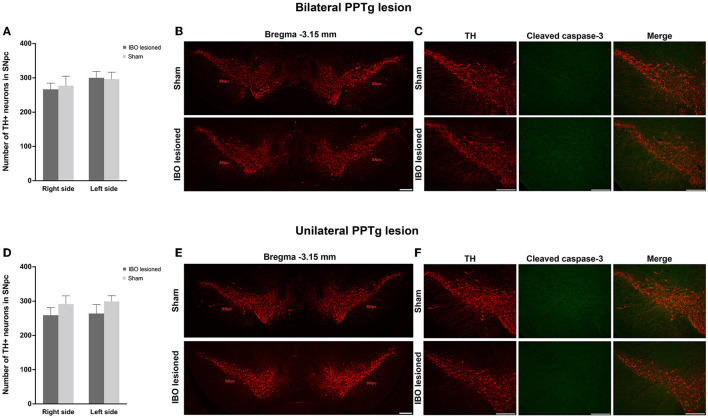
PPTg IBO lesion did not induce dopaminergic neuron loss and apoptosis in the SNpc. There was no change in the number of dopaminergic neurons **(A, B)** and apoptosis **(C)** in the SNpc following bilateral PPTg IBO lesion. In addition, there was no change in the number of dopaminergic neurons **(D, E)** and apoptosis **(F)** in the SNpc following unilateral PPTg IBO lesion. Scale bar = 1000 μm.

**Figure 7:**
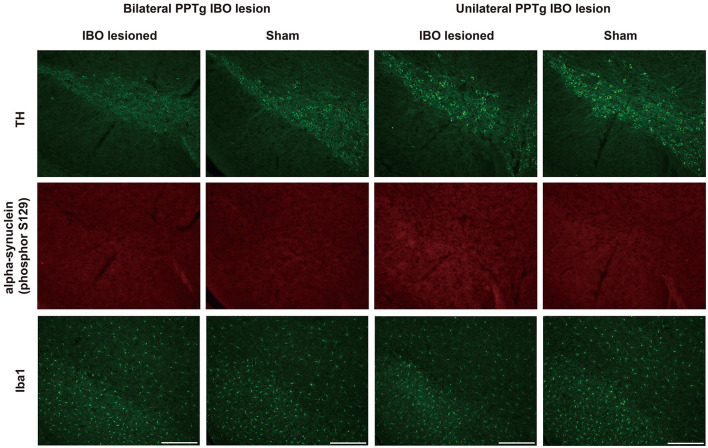
No neurodegeneration and microglial response were observed in the SNpc after the PPTg IBO lesion. Representative photographs of dopaminergic neurons, no fluorescence signal of alpha-synuclein (phosphor S129), and absent microglial response in the SNpc at AP −2.79 mm. Scale bar = 1000 μm.

Dystonia was featured by the imbalance of the dopaminergic and cholinergic system of the BG, in which dopaminergic neurons of the SNpc and cholinergic neurons of the CPu were mainly affected (34). Due to that, PPTg had abundant connections with the SNpc and CPu, and the loss of dopaminergic and cholinergic neurons was explored in the SNpc and CPu, respectively. For bilaterally lesioned mice, no loss of dopaminergic neurons was found for the left (lesioned: 300.00 ± 18.67, sham: 296.29 ± 3.06; *P* = 0.894) and right SNpc (lesioned: 266.63 ± 17.89, sham: 277.29 ± 27.61; *P* = 0.745; Figures 6A, B). No fluorescence signal of cleaved caspase-3 was found along the SNpc, reflecting no apoptosis in the SNpc (Figure 6C and Supplementary Figure S5). For unilaterally lesioned mice, there was also no loss of dopaminergic neurons in the left (lesioned: 264.00 ± 25.67, sham: 299.14 ± 16.86; *P* = 0.275) and right SNpc (lesioned: 258.86 ± 22.31, sham: 291.43 ± 24.22; *P* = 0.342; Figures 6D, E), and no apoptosis was found along the whole extent of the SNpc (Figure 6F). Moreover, no neurodegeneration and neuroinflammation were found in the SNpc because no fluorescence signal of alpha-synuclein (phosphor S129) and an absence of microglial response was detected in the lesioned mice (Figure 7 and Supplementary Figure S6).

**Figure 8:**
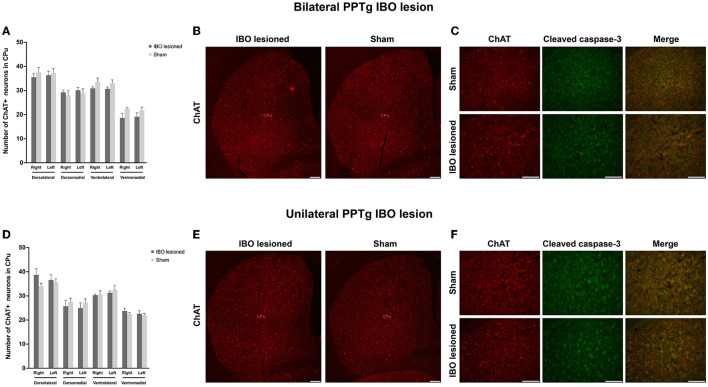
PPTg IBO lesion did not induce cholinergic neuron loss and apoptosis in the CPu. There was no change in the number of cholinergic neurons **(A, B)** and apoptosis **(C)** in the CPu following the bilateral PPTg IBO lesion. In addition, there was no change in the number of cholinergic neurons **(D, E)** and apoptosis **(F)** in the CPu following unilateral PPTg IBO lesion. Scale bar = 1000 μm. DL, dorsolateral; DM, dorsomedial; VL, ventrolateral; VM, ventromedial.

In addition, no significant loss of cholinergic neurons was found in the dorsolateral, dorsomedial, ventrolateral, and ventromedial parts of the bilateral CPu for bilaterally (Figures 8A, B and Supplementary Figure S7A) and unilaterally lesioned mice (Figures 8D, E and Supplementary Figure S7C), and no apoptosis was found in the dorsolateral part of the CPu (Figures 8C, F and Supplementary Figure S7B, D).

### Decreased neural activity in the BG and reticular formations following PPTg IBO lesions

**Figure 9:**
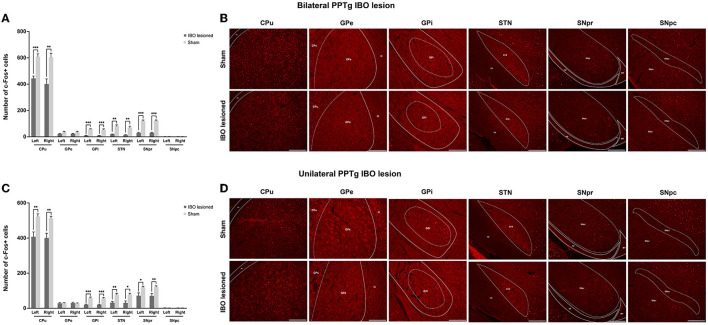
Alterations of the neural activity in the BG following the PPTg IBO lesion. **(A)** Significantly reduced neural activity was found in the dorsolateral part of the CPu, GPi, STN, and SNpr, while no change was observed for the GPe and SNpc after the bilateral lesion. **(B)** representative photographs of c-Fos+ cells in the dorsolateral part of the right CPu, GPi, STN, SNpr, and SNpc following the bilateral lesion. **(C)** Significantly reduced neural activity was found in the dorsolateral part of the CPu, GPi, STN, and SNpr, while no change was observed for the GPe and SNpc after the unilateral lesion. **(D)** Representative photographs of c-Fos+ cells in the dorsolateral part of the right CPu, GPe, GPi, STN, SNpr, and SNpc following the unilateral lesion. *P < 0.05, **P < 0.01, ***P < 0.001. Scale bar = 1000 μm.

**Figure 10:**
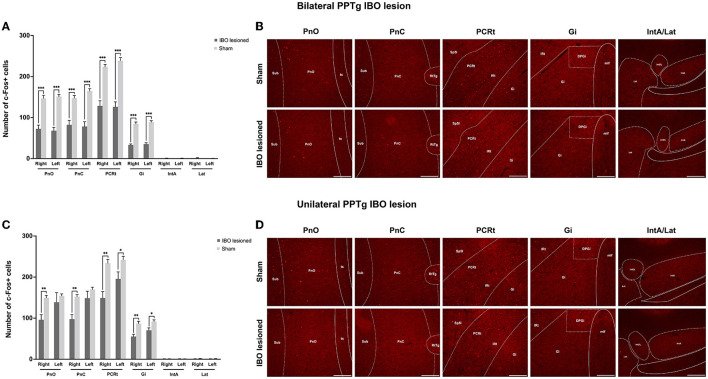
Alterations of the neural activity in the reticular formations and cerebellar structures following the PPTg IBO lesion. **(A)** Significantly reduced neural activity was found in the bilateral PnO, PnC, PCRt, and Gi, while no change was observed for the bilateral cerebellar nuclei of IntA and Lat after the bilateral lesion. **(B)** Representative photographs of c-Fos+ cells in the right PnO, PnC, PCRt, Gi, and IntA/Lat following the bilateral lesion. **(C)** Significantly reduced neural activity was found in the PnO (right), PnC (right), PCRt (bilateral), and Gi (bilateral), while no change was observed for the PnO (left), PnC (left), and bilateral cerebellar nuclei of IntA and Lat after the unilateral lesion. **(D)** representative photographs of c-Fos+ cells in the right PnO, PnC, PCRt, Gi, and IntA/Lat following the unilateral lesion. *P < 0.05, **P < 0.01, ***P < 0.001. Scale bar = 1000 μm.

With no structural deficit found for the SNpc and CPu, functional changes were explored for generating these abnormal behaviors. For bilaterally PPTg lesioned mice, there was significantly decreased neural activity in the dorsolateral part of the CPu (left, *P* < 0.001, Mann–Whitney *U*-test; right, *P* = 0.001, Mann–Whitney *U*-test), GPi (left, *P* < 0.001; right, *P* < 0.001), STN (left, *P* = 0.001; right, *P* = 0.002), and SNpr (left, *P* < 0.001; right, *P* < 0.001), while no change in neural activity was found for the GPe and SNpc (Figures 9A, B and Supplementary Figure S8). Moreover, the decrease of the c-Fos+ cells was significant in the lateral part of the SNpr. Similar results were found for the unilaterally lesioned mice, with a relatively milder change than for the bilaterally lesioned mice (Figures 9C, D). Regarding the reticular formations which received projections from the PPTg (Supplementary Figure S9), the neural activity of the PnO (left, *P* < 0.001; right, *P* < 0.001), PnC (left, *P* < 0.001; right, *P* < 0.001), PCRt (left, *P* < 0.001, Mann–Whitney *U*-test; right, *P* < 0.001), and Gi (left, *P* < 0.001; right, *P* < 0.001) were significantly reduced after bilateral PPTg lesions, while no change was found in the cerebellar nuclei including the IntA and Lat (Figures 10A, B and Supplementary Figure S10). In terms of the unilaterally lesioned mice, the neural activity of the right PnO (*P* = 0.004) and PnC (*P* = 0.001) were significantly reduced, while the left PnO (*P* = 0.209) and PnC (*P* = 0.273) showed no significant change. Bilateral PCRt (left, *P* = 0.033; right, *P* = 0.001) and Gi (left, *P* = 0.012; right, *P* = 0.004, Mann–Whitney *U*-test) showed significantly reduced neural activity after the unilateral lesion, with a predominance on the ipsilateral side to the lesion (Figures 10C, D and Supplementary Figure S10). However, c-Fos+ cells were rarely presented in the cerebellar nuclei of IntA and Lat in both lesioned and sham mice, and there was no significant change in the number of c-Fos+ cells in bilateral IntA and Lat after the unilateral PPTg lesion.

## Discussion

In this study, PPTg IBO lesioned mice presented dystonia-like behaviors such as joint flexion and toe clasping, and the dystonia behaviors were aggravated when the lesion affected bilateral PPTg. In addition, bilateral lesions induced impairments of proprioceptive and auditory sensory–motor integration, while unilaterally lesioned mice showed laterality of paw use and deficits in proprioceptive sensory–motor integration. Meanwhile, PPTg lesions did not impair motor learning, facial tactile sensory–motor integration, explorative behaviors, and gait. A remarkable loss of cholinergic neurons was found in the PPTg 21 days after the IBO lesion, and no loss of dopaminergic and cholinergic neurons was found in the SNpc and CPu, accompanied by no appearance of apoptosis, neuroinflammation, and neurodegeneration. However, after the PPTg IBO lesion, decreased neural activity was found in the BG as well as a wide range of reticular formations, which might contribute to these abnormal behaviors.

Studies have reported that toe clasping was commonly seen in mice with dystonia, followed by truncal twisting and forepaw clasping (35–37). For the genetic rodent model of dystonia, it is a fact that many models have lacked overt motor manifestations of typical dystonia (38). However, when genetic rodent models of dystonia were accompanied by explicit PPTg pathology changes, typical dystonia-like behaviors such as limb clasping, dystonic postures, and movements were noticed (12, 13). In our study, dystonia-like behaviors in our study were mainly due to muscle contractions of hindlimb flexors, reflecting the relatively elevated muscle tone of hindlimb flexors following the disruption of PPTg. With regard to the function of the PPTg in muscle tone, electrical stimulation of the PPTg could suppress the muscle tone, while the destruction of PPTg released musculature from inhibition and induced abnormal flexion of the spine and limbs in rats (17, 20, 39). In terms of the specific neural population of the PPTg, hindlimb clasping was observed in 100% of rats that underwent viral deposition of tau in cholinergic neurons in the bilateral PPTg (40). In addition, recent optogenetic studies found that different types of neurons in the PPTg had varied effects on muscle tone, with glutamatergic neurons in the PPTg preferably activated flexor muscles and cholinergic neurons activated extensor muscles (8, 21). Based on these, it was speculated that a nonspecific lesion of the PPTg could increase the muscle tone of hindlimbs, especially for flexor muscles. Furthermore, bilateral toe clasping and the abnormal flexion of the ankle and knee joint happened only in bilateral PPTg lesioned mice. With regard to these, it was assumed that toe flexors are modulated mainly by unilateral PPTg, while ankle and knee flexors are modulated by bilateral PPTg. Indeed, it was reported that the medullary reticular formation which dominated the input to the spinal cord and was involved in the modulation of locomotion and muscle tone received bilateral projections from the PPTg with an ipsilateral dominance, which might explain the laterality of dystonia found in our study (41).

Sensory information processing such as tactile, proprioceptive, and nociceptive information was found impaired in many types of dystonia, and it might contribute to the pathophysiology of motor dysfunction (16). Both bilaterally and unilaterally lesioned mice showed enhanced slips in the challenge beam test, while mice with a bilateral PPTg lesion showed overt deficits in the rotarod test. These deficits were in accordance with further findings of abnormal neural activities in the BG and reticular formations which were involved in the central selection of competing alternative actions and muscle tone control (6, 42). In addition, bilaterally lesioned mice showed a significantly reduced PPI of the acoustic startle response at 73 dB and 85 dB, while the unilateral mice did not. Early rat studies have found that a bilateral lesion of the PPTg could reduce PPI at startle intensity over 70 dB, which was in line with our results (43, 44). The negative results of the unilaterally lesioned mice might be explained by further findings that there was no significant change of the neural activity in the contralateral PnC in the unilaterally lesioned mice since there is evidence that the PPTg could modulate the acoustic startle reflex through the PnC (45).

In terms of the spontaneous explorative behaviors, bilateral PPTg lesioned mice showed decreased ambulatory episode average speed and vertical counts. A recent optogenetic study found that the photoactivation of glutamatergic neurons in the PPTg could increase the speed during ongoing locomotion, while the photoactivation of cholinergic or GABAergic neurons slowed or stopped ongoing locomotion (7). Another study found that the photoactivation of glutamatergic neurons significantly reduced the distance traveled (8). Based on these, we postulated that the glutamatergic neurons in the PPTg might be mainly affected in bilateral lesioned mice, which led to the decreased ambulatory speed and unchanged distance traveled. In terms of the reduced vertical counts, it was reported that there was an activation of c-Fos expression in the PPTg during upright posture in rats (46). Moreover, in patients with Parkinson's disease, deep brain stimulation of the PPTg could alleviate axial dystonia such as Pisa syndrome, which reflected the function of the PPTg in the vertical posture (47, 48).

From an anatomic perspective, the PPTg sent strong cholinergic and glutamatergic projections to the SNpc and modulated the activity of dopaminergic neurons and dopamine release in the striatum (49, 50). In addition, the pathological change of dopaminergic neurons in the SNpc might promote PPTg cell loss and activation of the PPTg in PD rats (51). Moreover, a recent study has found that the PPTg could send direct projections to the striatum and participate in the modulation of the activity of cholinergic interneurons and striatal spiny neurons (52, 53). Thus, it was speculated that the dystonia-like behaviors and abnormal sensory–motor integration were due to the pathological alterations of dopaminergic neurons in the SNpc and cholinergic interneurons in the CPu following a PPTg lesion. However, no neural loss, apoptosis, and neurodegeneration were identified for these neurons in our study. It might be due to the relatively short duration between the lesion and the sacrifice, which could not induce these pathological changes in time. Indeed, the loss of dopaminergic neurons in the SNpc was found after bilateral cholinergic PPTg lesions for 7 weeks in rats, while no significant loss of dopaminergic neurons was noted when the lesion duration decreased to 3 weeks (54).

Although no structural and neurodegenerative changes were identified in the SNpc and CPu, the neural activity of the CPu, GPi, STN, and SNpr was decreased following either a bilateral or unilateral PPTg lesion. The GPi, STN, and SNpr receive monosynaptic excitatory projections from the PPTg, and the GPi and SNpr also receive excitatory projections from the STN, which constitutes the striatal indirect pathway of the BG (55). The function of the indirect pathway modulates the inhibition of competing actions during locomotion, and the hypoactivity of the indirect pathway was an important feature of dystonia (22, 56). A PPTg lesion was thought to induce the hypoactivity of the STN and SNpr, and it was confirmed that a unilateral lesion of the PPTg could reverse the hyperactivity of the STN and SNpr in a 6-hydroxydopamine rat model (57). Moreover, a recent neuroanatomical study showed that the lateral part of the SNpr mainly received indirect pathway-related innervations (58). Combined with our finding that the neural activity of the lateral part of the SNpr was significantly reduced, it was proposed that the indirect pathway was impaired. In addition, the PPTg could send projections to the SNpc and CPu, which might thereafter affect the imbalance of the direct and indirect pathways. However, the effect on the SNpc was ruled out because both lesioned and sham mice showed very weak activation. In terms of the hypoactivity of the dorsolateral part of the CPu found in our study, recent studies have shown that the activation of cholinergic or glutamatergic inputs from the PPTg to the CPu could induce activation of cholinergic interneurons and inhibition of striatal spiny neurons (53, 59). Thus, after a PPTg lesion, the hypoactivity of the CPu might be due to the decreased neural activity of cholinergic interneurons or the impaired indirect pathway.

With regard to reticular formations, the PPTg caused hypoactivity of PnO, PnC, PCRt, and Gi. These reticular formations mainly contain inhibitory GABA/glycinergic neurons which directly inhibit muscle tone (6). In a recent study, the photoactivation of GABAergic neurons in the PCRt abolished muscle activity was seen (60). Based on this, a PPTg lesion might induce dystonia-like clasping and joint flexion directly through the inactivation of inhibitory neurons in reticular formations. In addition, although the cerebellum is affected in many types of dystonia and has connections with the PPTg (5), we did not find alterations of neural activity in the cerebellar structure, which indicated that the abnormal behaviors following the PPTg lesion were mainly related to its effect on the BG and reticular formations.

There were some limitations in this study. First, although a working definition of dystonia was postulated as any abnormal twisting movements in rodents (4), there is still uncertainty regarding the dystonia manifestation of mice mimicking the presentation and the mechanism of dystonia in human beings. The co-contraction of agonists and antagonists might be an important electrophysiological feature, but it lacked specificity because it could also present in normal joint stiffening (61). In our study, dystonia-like behaviors were evaluated and analyzed meticulously according to the dystonia manifestations presented in previous mice models of dystonia. Combined with previous reports of the involvement of the PPTg in patients and mice with genetic dystonia, it might reflect a relationship between the PPTg and dystonia. Second, all the experimental mice were sacrificed 21 days after a lesion was induced. With the relatively short duration, dystonia-like behaviors could only be explained by the functional changes of the PPTg-related motor regions. Although persisted hindlimb clasping was found in a rat that underwent bilateral tau expression in cholinergic neurons of the PPTg for 20 weeks (40), studies with a longer PPTg lesion duration should be conducted for the exploration of the dystonia-like behaviors and its underlying mechanism.

## Conclusion

Our study showed that a nonspecific lesion of the PPTg could induce dystonia-like behaviors and impair sensory–motor integration, which was in accordance with the previous finding that the PPTg might participate in the development of dystonia. In addition, these abnormal behaviors were mainly related to the functional changes of the striatal indirect pathway and reticular formations rather than structural changes in the BG such as apoptosis, glia response, and neurodegeneration.

## Data availability statement

The raw data supporting the conclusions of this article will be made available by the authors, without undue reservation.

## Ethics statement

The animal study was reviewed and approved by the Institutional Animal Care and Use Committee of Tongji University.

## Author contributions

J-HS: conceptualization, original draft, and revising. Y-WH: formal analysis and visualization. Y-PS: conceptualization and reviewing. YY: formal analysis and data curation. R-YL: revising. K-GZ and LH: methodology. X-HW: reviewing and revising. FT: reviewing and supervision. L-JJ: supervision, revising, and editing of the final version of the manuscript. All authors contributed to the article and approved the submitted version.

## Supplementary material

Supplementary Video 1 Dystonia-like behaviors of mice following the bilateral and unilateral PPTg IBO lesion at day 8 and 15.
