# Supplementary material for: Dystonia-like behaviors and impaired sensory–motor integration following neurotoxic lesion of the pedunculopontine tegmental nucleus in mice
Source: Front Neurol. 2023 Mar 30;14:1102837. doi: 10.3389/fneur.2023.1102837 (PMC10101329; doi:10.3389/fneur.2023.1102837)
Supplement: Supplementary file 2 [file Data_Sheet_1.docx]

**Supplementary tables**

**Table S1. Dystonia score of tail suspension test for mice underwent bilateral and unilateral PPTg IBO lesion**

|  |  | **Dystonia score** | | | | | | | | | |
| --- | --- | --- | --- | --- | --- | --- | --- | --- | --- | --- | --- |
|  |  | **Day 8** | | | |  | **Day 15** | | | | |
|  |  | **Hindlimb** | **Forelimb** | **Trunk** | **Total** |  | **Hindlimb** | **Forelimb** | **Trunk** | **Total** | |
| 11 | BI-IBO | 1 | 1 | 0 | 2 |  | 1 | 1 | 0 | 2 | |
| 13 | BI-IBO | 2 | 1 | 0 | 3 |  | 1 | 0 | 0 | 1 | |
| 21 | BI-IBO | 3 | 1 | 0 | 4 |  | 3 | 0 | 0 | 3 | |
| 22 | BI-IBO | 1 | 0 | 0 | 1 |  | 1 | 0 | 0 | 1 | |
| 23 | BI-IBO | 2 | 1 | 0 | 3 |  | 2 | 1 | 0 | 3 | |
| 24 | BI-IBO | 2 | 0 | 1 | 3 |  | 2 | 0 | 1 | 3 | |
| 25 | BI-IBO | 2 | 0 | 1 | 3 |  | 2 | 0 | 0 | 2 | |
| 43 | BI-IBO | 2 | 0 | 1 | 3 |  | 1 | 0 | 0 | 1 | |
| 32 | BI-Sham | 0 | 0 | 0 | 0 |  | 1 | 0 | 0 | 1 | |
| 33 | BI-Sham | 0 | 0 | 0 | 0 |  | 0 | 0 | 0 | 0 | |
| 34 | BI-Sham | 0 | 0 | 0 | 0 |  | 0 | 0 | 0 | 0 | |
| 35 | BI-Sham | 0 | 0 | 0 | 0 |  | 0 | 0 | 0 | 0 | |
| 45 | BI-Sham | 0 | 0 | 0 | 0 |  | 0 | 0 | 0 | 0 | |
| 54 | BI-Sham | 0 | 0 | 0 | 0 |  | 0 | 0 | 0 | 0 | |
| 55 | BI-Sham | 2 | 0 | 0 | 2 |  | 0 | 0 | 0 | 0 | |
| 14 | UNI-IBO | 2 | 1 | 0 | 3 |  | 2 | 0 | 0 | 2 | |
| 26 | UNI-IBO | 1 | 0 | 0 | 1 |  | 0 | 0 | 0 | 0 | |
| 31 | UNI-IBO | 2 | 0 | 0 | 2 |  | 1 | 0 | 0 | 1 | |
| 41 | UNI-IBO | 0 | 0 | 1 | 1 |  | 2 | 0 | 0 | 2 | |
| 42 | UNI-IBO | 1 | 0 | 0 | 1 |  | 0 | 0 | 0 | 0 | |
| 44 | UNI-IBO | 0 | 1 | 0 | 1 |  | 0 | 0 | 0 | 0 | |
| 51 | UNI-IBO | 2 | 0 | 0 | 2 |  | 2 | 0 | 1 | 3 | |
| 61 | UNI-Sham | 1 | 0 | 0 | 1 |  | 1 | 0 | 0 | 1 | |
| 62 | UNI-Sham | 0 | 0 | 0 | 0 |  | 0 | 0 | 0 | 0 | |
| 63 | UNI-Sham | 0 | 0 | 0 | 0 |  | 0 | 0 | 0 | 0 | |
| 64 | UNI-Sham | 0 | 0 | 0 | 0 |  | 0 | 0 | 0 | 0 | |
| 65 | UNI-Sham | 0 | 0 | 0 | 0 |  | 0 | 0 | 0 | 0 | |
| 66 | UNI-Sham | 0 | 0 | 0 | 0 |  | 0 | 0 | 0 | 0 | |
| 67 | UNI-Sham | 0 | 0 | 0 | 0 |  | 0 | 0 | 0 | 0 | |
| BI, bilateral; UNI, unilateral. | | | | | | | | | | |  |

**Supplementary figures**

**
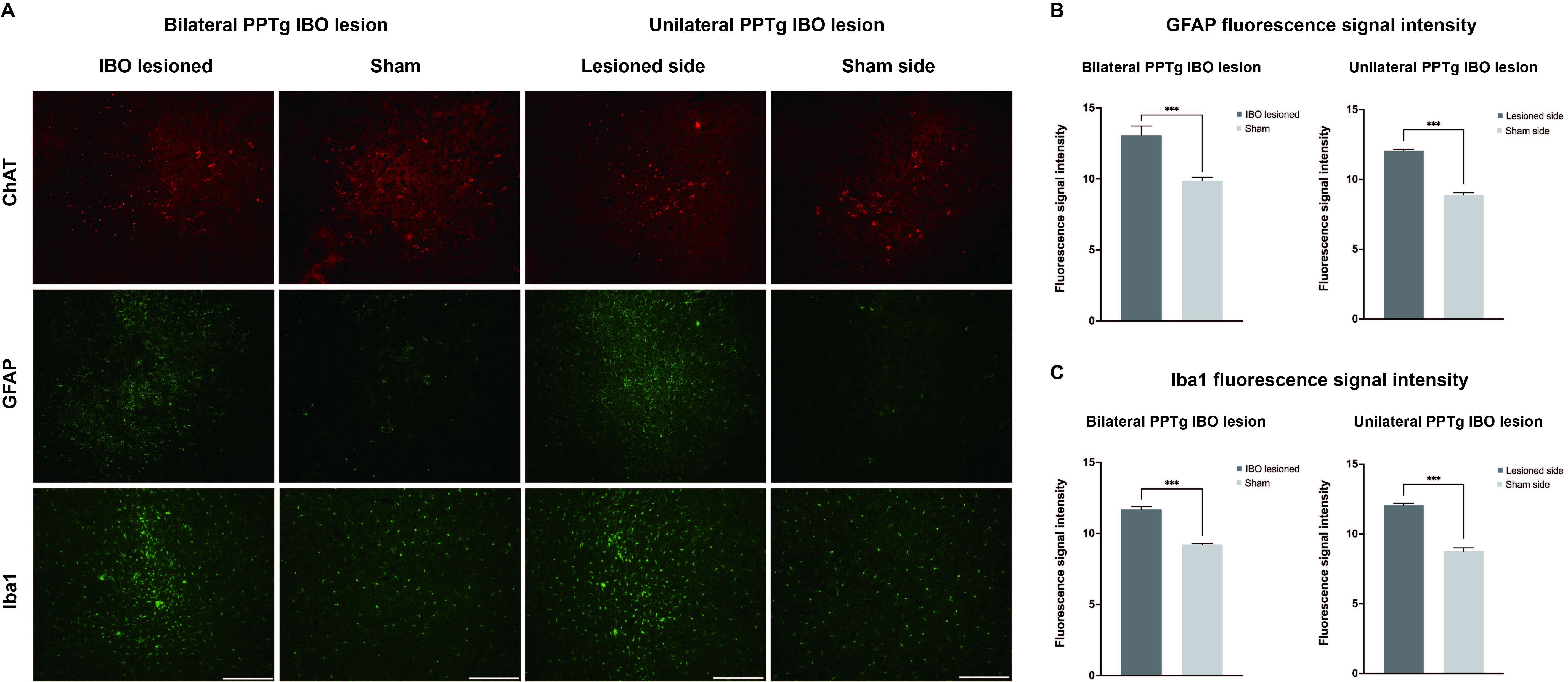
**

**Fig S1. Loss of ChAT+ neurons and local glial responses nearby the infusion area after bilateral and unilateral PPTg IBO lesion.** (A) representative photographs of loss of ChAT+ neurons, enhanced astroglial and microglial responses nearby the infusion area at AP -4.74 mm. (B) Significantly enhanced fluorescence signal intensity of GFAP was found in PPTg lesioned mice compared with sham mice. (C) Significantly enhanced fluorescence signal intensity of Iba1 was found in PPTg lesioned mice compared with sham mice. * *P* < 0.05, ** *P* < 0.01, *** *P* < 0.001. Scale bar = 1000 μm.

**
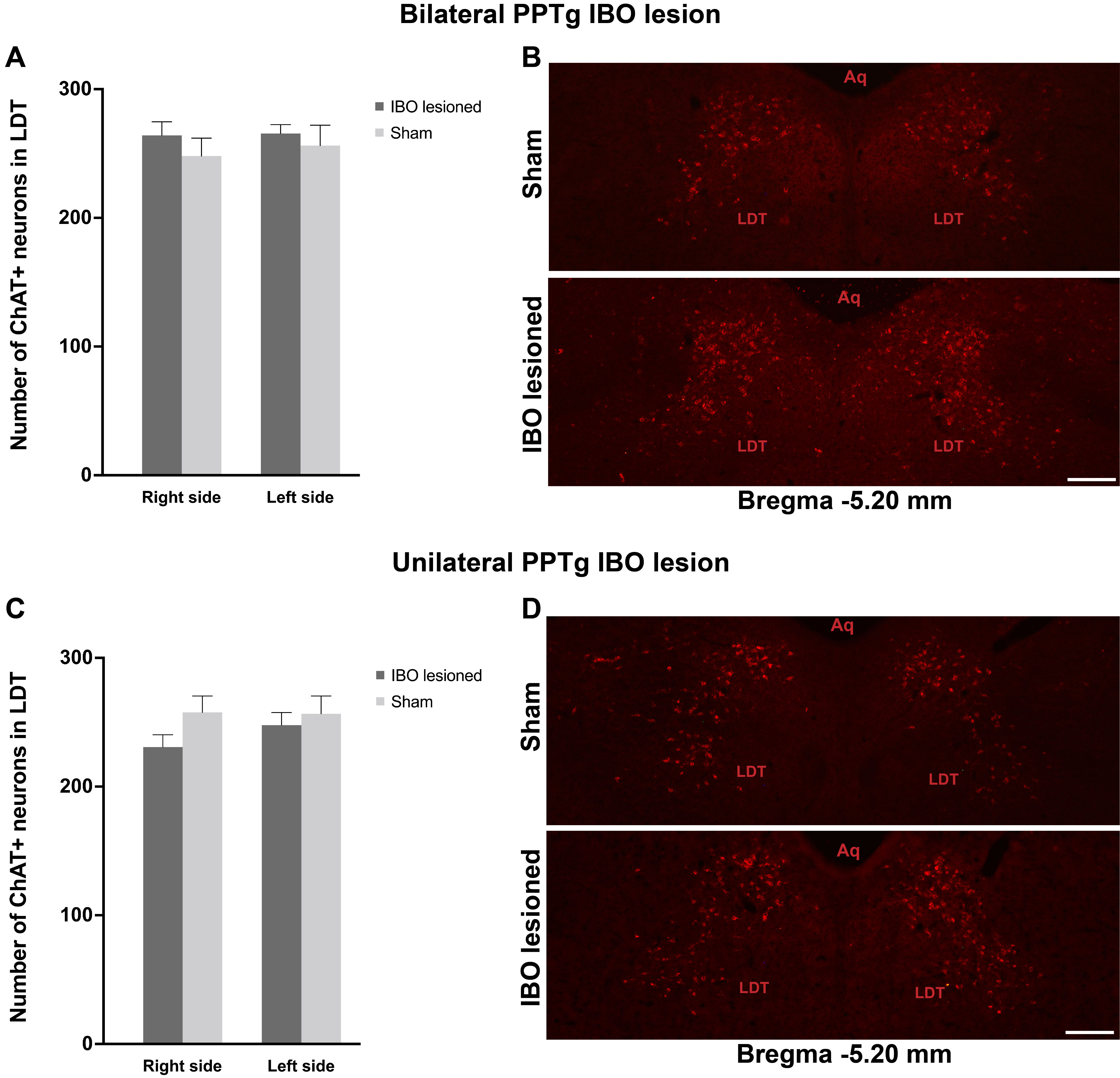
**

**Fig S2. Quantification of cholinergic neurons and lesion analysis in the LDT for mice underwent bilateral and unilateral PPTg IBO lesion.** (A) the comparison of absolute number of cholinergic neurons in the LDT between bilateral IBO lesioned and sham lesioned mice. (B) representative photographs of ChAT staining of the LDT (at AP -5.20 mm) for bilateral IBO lesioned and sham lesioned mice. (C) the comparison of absolute number of cholinergic neurons in the LDT between unilateral IBO lesioned and sham lesioned mice. (D) representative photographs of ChAT staining of the LDT (at AP -5.20 mm) for unilateral IBO lesioned and sham lesioned mice. Scale bar = 1000 μm.





**Fig S3. No overt deficit was found for PPTg IBO lesioned mice in the open field test.** For both bilateral and unilateral PPTg IBO lesioned mice, there was no difference on the ambulatory distance (A), ambulatory counts (C), stereotypic counts (D) and jump counts (F) between them and the sham groups at day 9 and day 16. Bilateral PPTg lesioned mice showed lower ambulatory episodes average speed at day 16 and vertical counts at day 9 (B, E) than the sham group, while no significant changes of them were seen in the unilateral PPTg lesioned mice (B, E;). * *P* < 0.05, ** *P* < 0.01, *** *P* < 0.001.


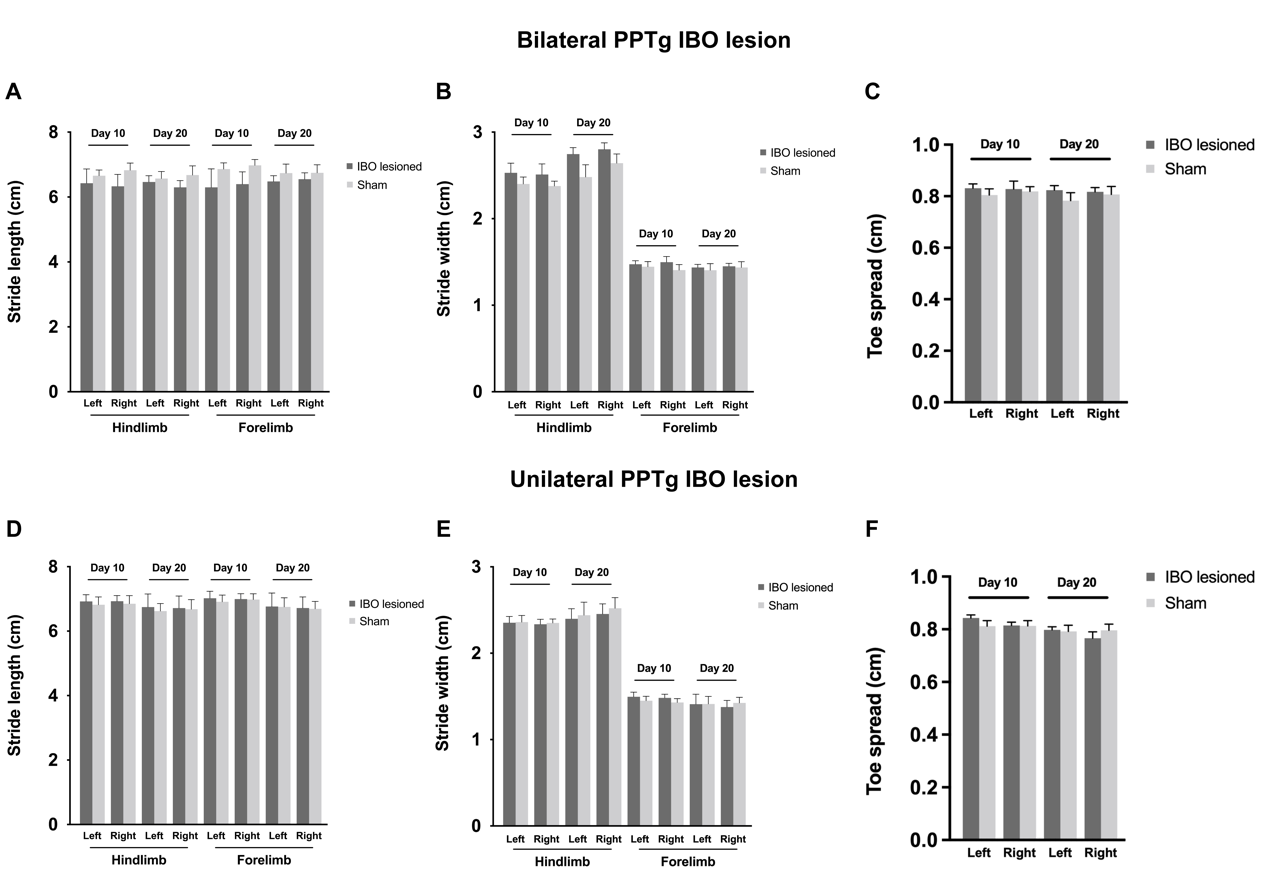


**Fig S4. No significant impairment was found for PPTg IBO lesioned mice in the footprint test.** Mice underwent bilateral PPTg IBO lesion showed insignificantly shorter and stride length and higher stride width of forelimb and hindlimb at day 10 and day 20 (A, B), while no difference was found for the unilateral lesioned mice (D, E). In addition, no changes of toe spread (C, F) were found between PPTg lesioned mice and the sham mice at day 9 and day 16. * *P* < 0.05, ** *P* < 0.01, *** *P* < 0.001.


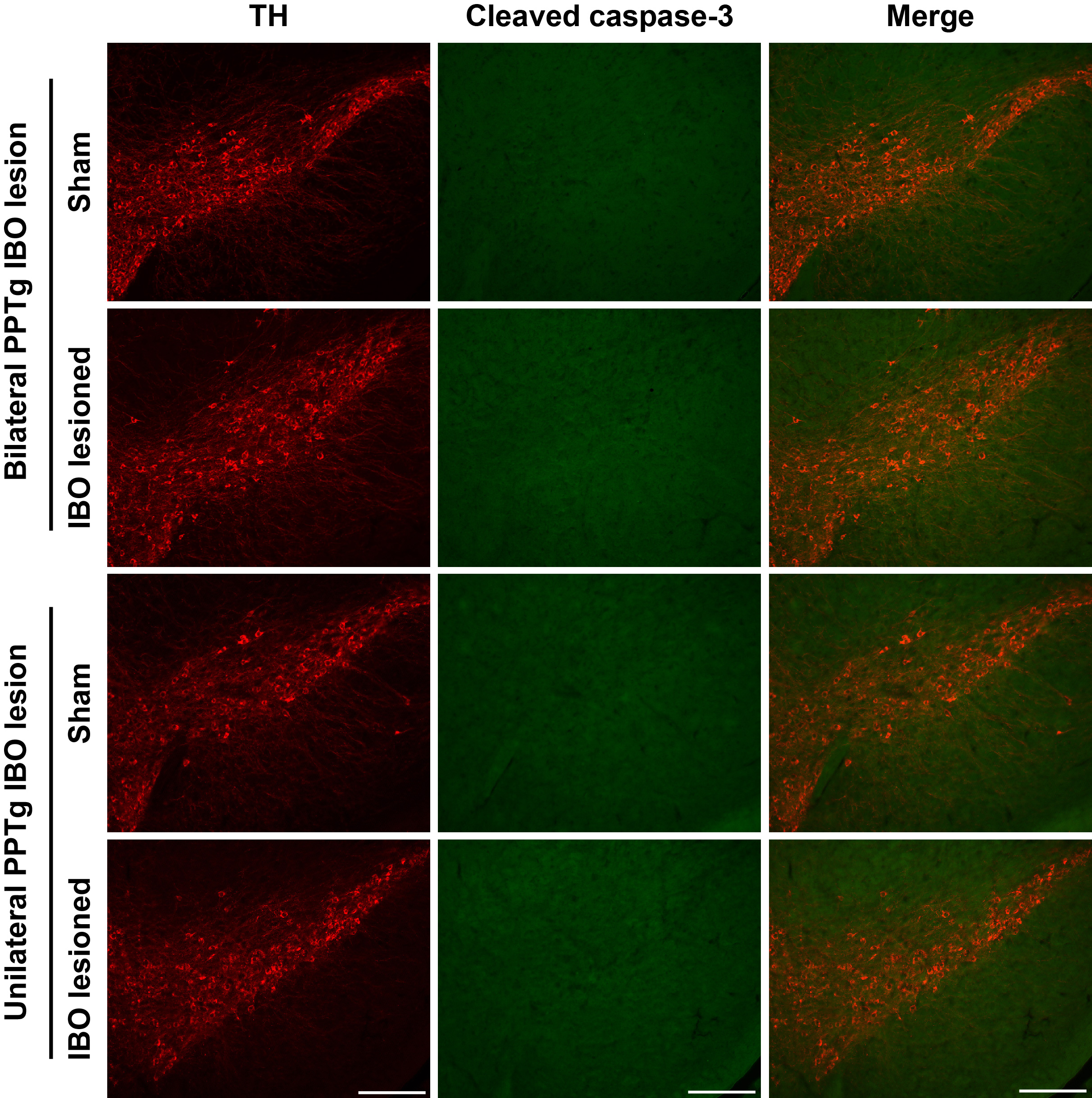


**Fig S5. No dopaminergic neuron loss and apoptosis was found in the left SNpc of mice with bilateral and unilateral PPTg IBO lesion.** Representative photographs of dopaminergic neurons, no fluorescence signal of cleaved caspase-3 in the left SNpc at AP -2.79 mm. Scale bar = 1000 μm.


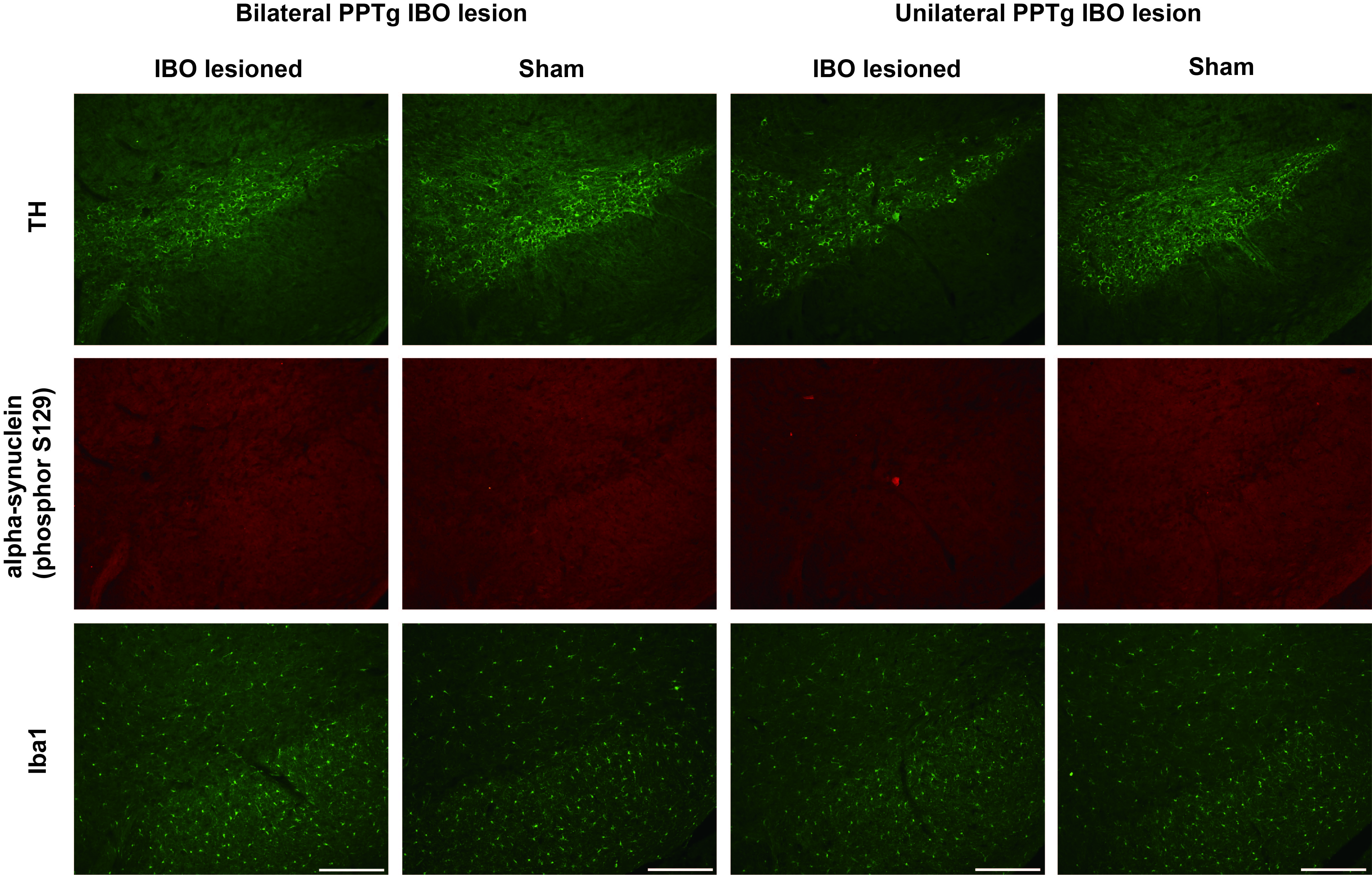


**Fig S6. No neurodegeneration and microglial response were observed in the left SNpc after PPTg IBO lesion.** Representative photographs of dopaminergic neurons, no fluorescence signal of alpha-synuclein (phosphor S129) and absent microglial response in the left SNpc at AP -2.79 mm. Scale bar = 1000 μm.


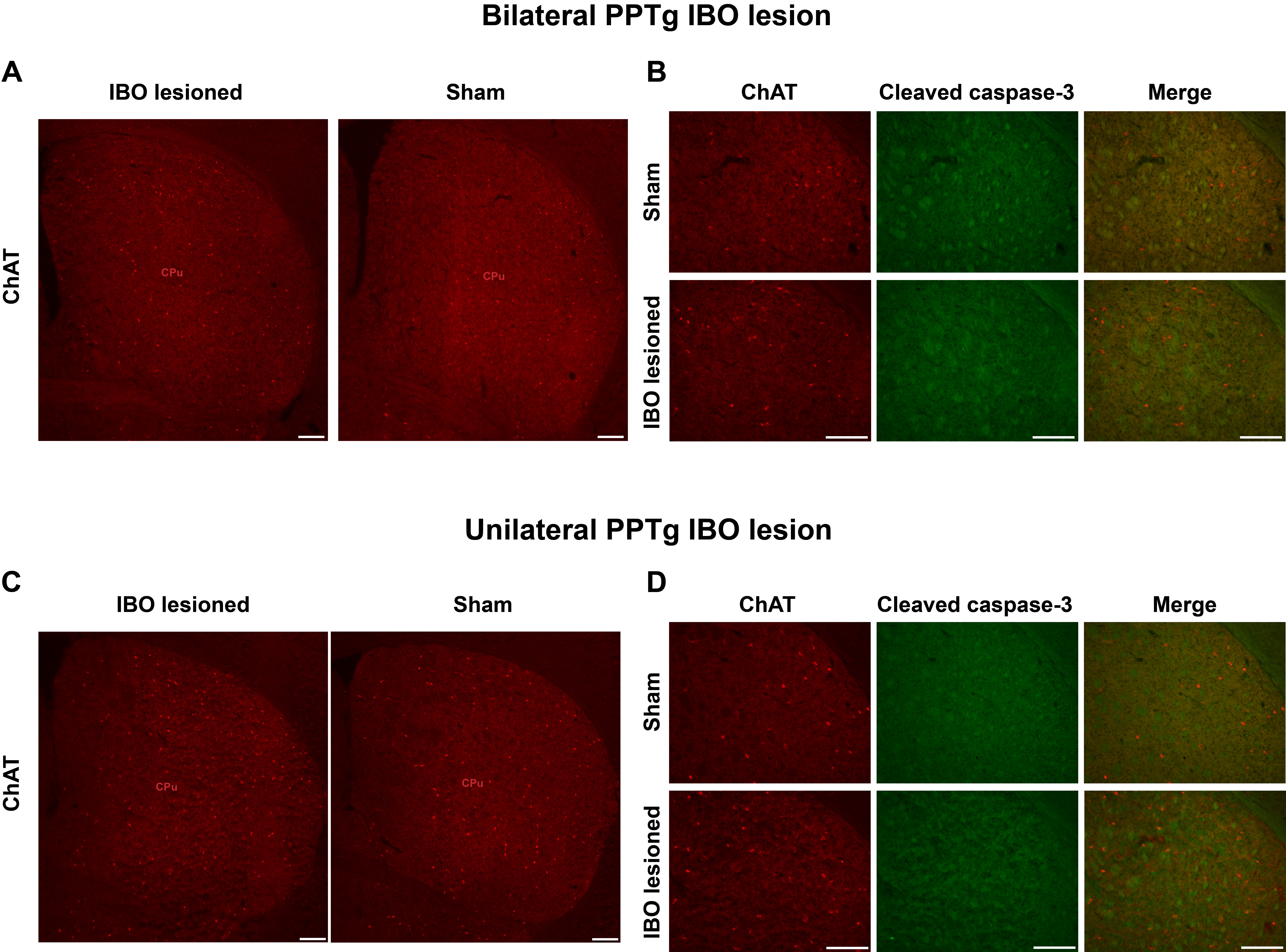


**Fig S7. PPTg IBO lesion did not induce cholinergic neuron loss and apoptosis in the CPu.** No significant loss and apoptosis of cholinergic neurons were found in the left CPu following bilateral PPTg IBO lesion (A, B). In addition, no significant loss and apoptosis of cholinergic neurons were found in the left CPu following unilateral PPTg IBO lesion (C, D). Scale bar = 1000μm.

**
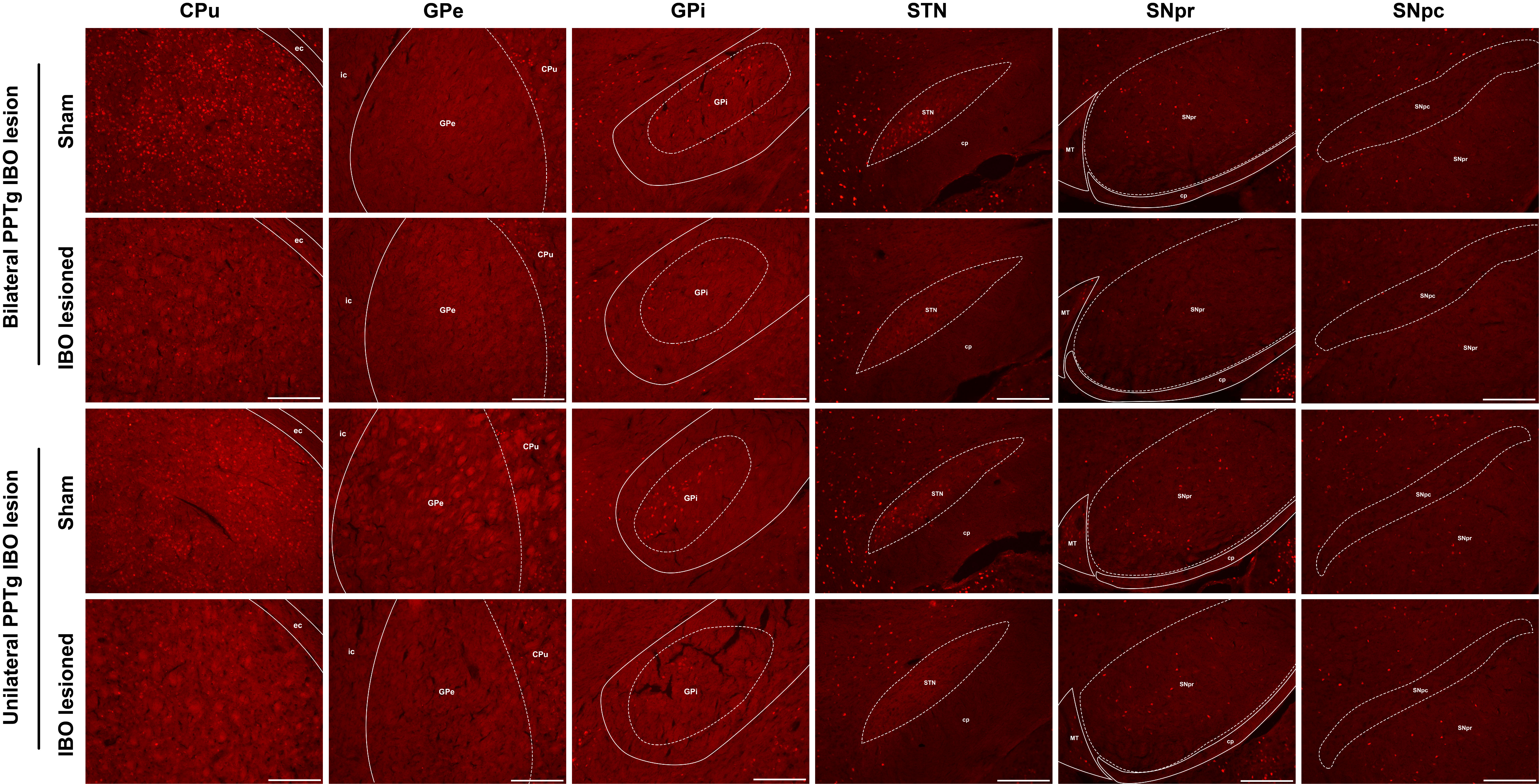
Fig S8. Alterations of the neural activity in the BG following the PPTg IBO lesion.** Representative photographs of reduced c-Fos+ cells in the dorsolateral part of the left CPu, GPi, STN and SNpr, while no change was observed for the GPe and SNpc after the bilateral and unilateral lesion. Scale bar = 1000μm.


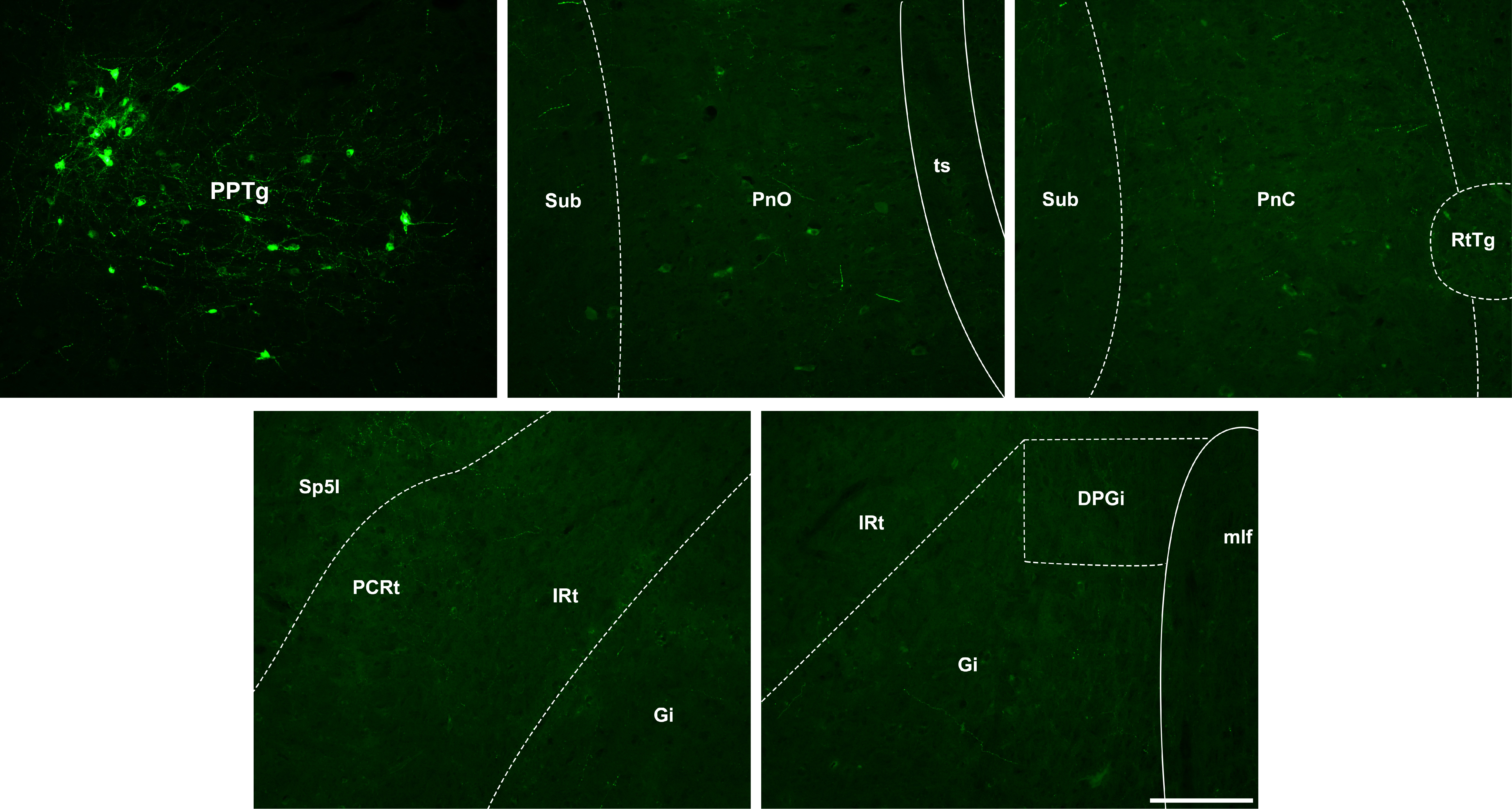


**Fig S9. Preliminary tracing results of reticular formations which received the PPTg cholinergic projection.** ChAT Cre mice (n=3) received 200 nL of rAAV-EF1a-DIO-EGFP (5.14 × 10^12^ genomic copies/mL; BrainCase Co., Ltd., Shenzhen, China) injection for the right PPTg (at AP: -4.60 mm, ML: +1.20 mm and DV: -3.70 mm). After 30 days, strong positive GFP signals were detected in the cholinergic neurons of the right PPTg, and cholinergic projections which expressed EGFP were observed predominantly in the PnO, PnC, PCRt and Gi ipsilateral to the injection. Scale bar = 1000 μm.


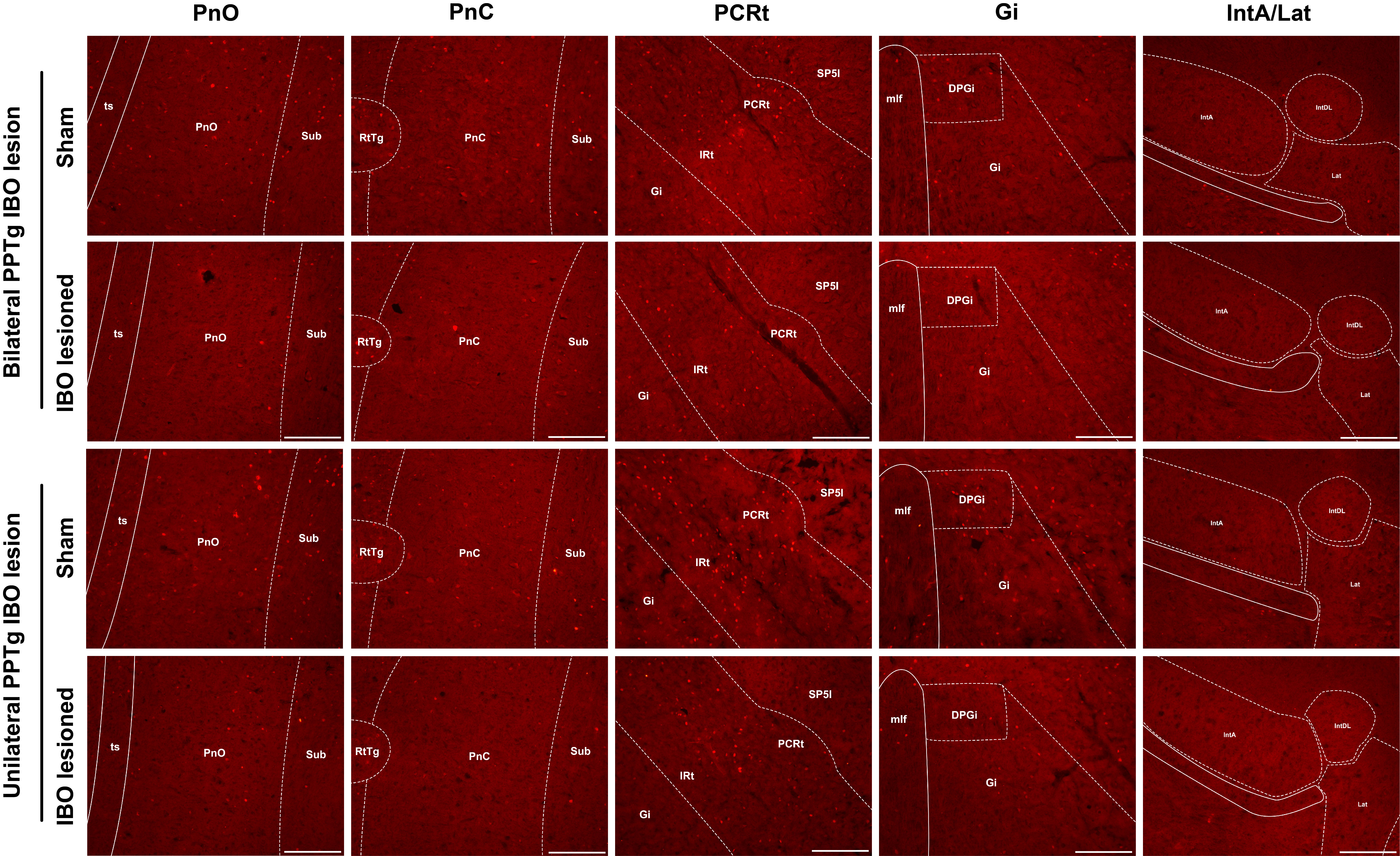


**Fig S10. Alterations of the neural activity in the reticular formations and cerebellar structures following the PPTg IBO lesion.** Representative photographs of c-Fos+ cells in the left PnO, PnC, PCRt, Gi and IntA/Lat following the bilateral and unilateral lesion. Scale bar = 1000 μm.
